# Acute Sarcopenia: Mechanisms and Management

**DOI:** 10.3390/nu16203428

**Published:** 2024-10-10

**Authors:** Sarah Damanti, Eleonora Senini, Rebecca De Lorenzo, Aurora Merolla, Simona Santoro, Costanza Festorazzi, Marco Messina, Giordano Vitali, Clara Sciorati, Patrizia Rovere-Querini

**Affiliations:** 1 Internal Medicine Unit, IRCCS San Raffaele Scientific Institute, 20132 Milan, Italy; damanti.sarah@hsr.it (S.D.); vitali.giordano@hsr.it (G.V.); rovere.patrizia@hsr.it (P.R.-Q.); 2 Division of Immunology, Transplantation and Infectious Diseases, Vita-Salute San Raffaele University, 20100 Milan, Italy; senini.eleonora@hsr.it (E.S.); delorenzo.rebecca@hsr.it (R.D.L.); merolla.aurora@hsr.it (A.M.); santoro.simona@hsr.it (S.S.); festorazzi.costanza@hsr.it (C.F.); messina.marco@hsr.it (M.M.)

**Keywords:** acute sarcopenia, muscle wasting, hospitalization

## Abstract

Background: Acute sarcopenia refers to the swift decline in muscle function and mass following acute events such as illness, surgery, trauma, or burns that presents significant challenges in hospitalized older adults. Methods: narrative review to describe the mechanisms and management of acute sarcopenia. Results: The prevalence of acute sarcopenia ranges from 28% to 69%, likely underdiagnosed due to the absence of muscle mass and function assessments in most clinical settings. Systemic inflammation, immune–endocrine dysregulation, and anabolic resistance are identified as key pathophysiological factors. Interventions include early mobilization, resistance exercise, neuromuscular electrical stimulation, and nutritional strategies such as protein supplementation, leucine, β-hydroxy-β-methyl-butyrate, omega-3 fatty acids, and creatine monohydrate. Pharmaceuticals show variable efficacy. Conclusions: Future research should prioritize serial monitoring of muscle parameters, identification of predictive biomarkers, and the involvement of multidisciplinary teams from hospital admission to address sarcopenia. Early and targeted interventions are crucial to improve outcomes and prevent long-term disability associated with acute sarcopenia.

## 1. Introduction

Acute sarcopenia is characterized by a rapid decline in muscle mass and function, typically triggered by acute events such as illness, surgery, trauma, or hospitalization. While chronic sarcopenia associated with aging has been extensively studied, acute sarcopenia remains a relatively underexplored area, despite its significant impact on patient outcomes, especially in older adults. The swift onset of muscle degradation in response to acute stressors poses unique diagnostic and therapeutic challenges, often leading to prolonged recovery, increased risk of complications, and higher mortality rates.

This narrative review aims to provide a comprehensive examination of the mechanisms, clinical implications, and management strategies for acute sarcopenia. Specifically, the objectives of this review are to: (i) summarize the current understanding of the pathophysiological mechanisms driving acute sarcopenia, (ii) discuss the diagnostic challenges and the limitations of current methods used to assess muscle mass and function in acutely ill patients, (iii) evaluate the existing therapeutic interventions, such as nutritional supplementation, physical therapy, and pharmacological agents, while highlighting the variable efficacy of these treatments in preventing or mitigating muscle loss, (iv) identify key gaps in the literature and propose future research directions, particularly in the areas of early intervention, biomarker identification, and personalized treatment approaches for acute sarcopenia, (v) emphasize the importance of multidisciplinary management, integrating medical, nutritional, and rehabilitative strategies to improve outcomes for patients at risk of or recovering from acute sarcopenia. By addressing these objectives, this review aims to provide healthcare professionals and researchers with a clearer understanding of acute sarcopenia and its clinical significance, while also highlighting the urgent need for further investigation and evidence-based interventions.

## 2. Methods

Given the narrative nature of this review, the literature search was conducted with the aim of providing a comprehensive overview of current knowledge on acute sarcopenia. Peer-reviewed journals were consulted to cover the key aspects of the condition, from underlying mechanisms to therapeutic interventions. The search was carried out using PubMed, focusing on articles published in the last decade, although older foundational studies were also included when relevant. Search terms included “acute sarcopenia”, “muscle wasting”, “hospitalization-related muscle loss”, “acute sarcopenia treatment”. No rigid inclusion or exclusion criteria were applied, given that the objective of a narrative review is to synthesize and discuss available literature rather than perform a systematic assessment. Instead, studies were selected based on their relevance to the understanding of acute sarcopenia’s pathophysiology, diagnosis, and management. Through this approach, the review captures the breadth of the topic and offers insights into the current state of knowledge while identifying areas where further research is needed.

## 3. Definitions of Sarcopenia

**Figure 1:**
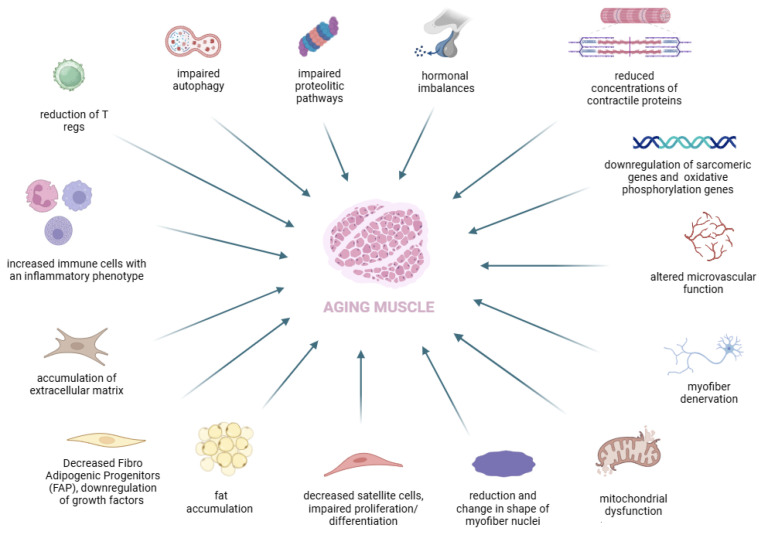
Main modifications in aging muscles.

Sarcopenia is a disease characterized by a reduction of muscle mass and function which is associated with an increased risk of developing adverse clinical outcomes [1]. Primary sarcopenia is a chronic condition; it refers to the gradual decline of muscle function and mass typical of aging [2]. Muscle mass decreases on average from 50% of total body weight in young adults to 25% in those over 80 years old [3]. The mechanisms driving this process are complex and multifactorial, and they are not yet completely understood [4]. Disrupted protein homeostasis, impaired proteolytic and autophagic pathways [5,6,7,8], mitochondrial dysfunction [9], reduction in myofiber nuclei [10,11], decrease in satellite cell numbers and/or alterations in their proliferation and differentiation abilities [12,13], myofiber denervation [14], diminished and altered microvascular structure [15], elevated levels of inflammatory mediators [16], and hormonal imbalances [17] are all significant contributors to the age-related decline in muscle mass and function (Figure 1).

This figure presents a schematic overview of the key factors contributing to muscle degeneration in aging. The central image depicts aging muscle surrounded by various pathological changes that impact muscle health, like impaired autophagy, reduction in the muscle’s ability to degrade and recycle damaged proteins, leading to accumulation of dysfunctional proteins, decreased levels of hormones like testosterone and growth hormone, along with a decline in muscle proteins and downregulation of sarcomeric and oxidative phosphorylation genes, contributing to muscle weakness and atrophy. Increased presence of immune cells with an inflammatory profile and excessive extracellular matrix deposition and fat accumulation can impair muscle function and regeneration. Moreover, decreased mitochondrial function reduces energy production, while loss of nerve innervation leads to muscle fiber atrophy. These factors collectively highlight the multifaceted nature of muscle aging, which is exacerbated in conditions like sarcopenia.

In particular, inflammation and denervation can alter myonuclear identity with type I myonuclei undergoing a metabolic shift towards a more glycolytic profile [3]. On the contrary, type II myonuclei display an important glycogen depletion, making them more vulnerable to muscle atrophy [3]

When sarcopenia is caused by factors beyond the natural aging process, such as diseases, inactivity, or malnutrition, it is classified as secondary sarcopenia. For many older adults, sarcopenia arises from a combination of these factors, making it challenging to categorize each case strictly as primary or secondary.

Muscle strength is the key indicator of sarcopenia, as it currently serves as the most reliable measure of muscle function. Sarcopenia is probable when low muscle strength is observed. To confirm a diagnosis of sarcopenia, there must also be evidence of reduced muscle mass or quality. When low muscle strength, diminished muscle quantity or quality, and reduced physical performance are all present, sarcopenia is classified as severe. Table 1 illustrates the cut-off to diagnose sarcopenia according to the European Working Group on Sarcopenia in Older People 2 definition [2], the 2019 Asian Working Group for Sarcopenia [18], and the Sarcopenia Definition and Outcomes Consortium [19].

Acute sarcopenia is characterized by a rapid onset of muscle insufficiency following illness, surgery, trauma, or burns. There is no consensus on the period during which the onset of sarcopenia can be considered related to an acute event, with some studies using a 28-day cut-off [20,21] and others using 6 months [2,22]. To define acute sarcopenia, the decline in muscle mass and function must be significant enough to meet the criteria for sarcopenia using previously defined cut-off points (Table 1) [1].

**Figure 2:**
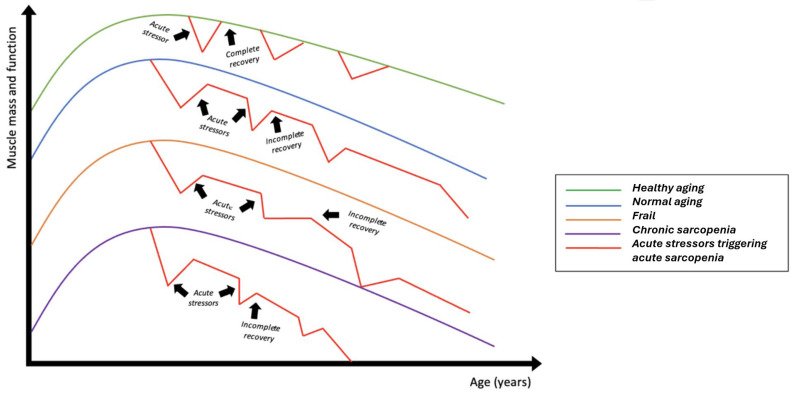
Patterns of acute and chronic sarcopenia throughout the lifespan.

Some individuals experience an acute-on-chronic phenotype, where acute illnesses exacerbate chronic sarcopenia. This condition is termed acute-on-chronic sarcopenia [23]. Recovery from acute sarcopenia can be incomplete (Figure 2), especially in frail and older adults [24,25], who may not return to their pre-illness baseline muscle status [20].

This figure illustrates the progression of muscle mass and function over time under various aging conditions, emphasizing the impact of acute stressors: (i) healthy aging: represented by a green line, showing gradual decline in muscle mass and function with occasional stressors leading to complete recovery. (ii) Normal aging: shown in blue, with a more pronounced decline and less robust recovery following acute stressors. (iii) Frail: indicated by the orange line, depicting accelerated decline with incomplete recovery after stressors, leading to persistent functional impairment. (iv) Chronic sarcopenia: marked by the purple line, characterized by a steep decline in muscle mass and function with minimal recovery, highlighting the vulnerability of individuals with sarcopenia to acute stressors. The figure underscores the importance of maintaining muscle health to mitigate the impact of aging and acute health events on muscle function.

The accumulation of short periods of acute sarcopenia throughout an individual’s lifespan likely contributes significantly to the etiology of chronic sarcopenia [26]. With an assumption of 80% recovery of muscle mass following each period of disuse, an older person would lose at least 400 g of muscle tissue after only two short periods of illness or injury per year. This equates to a 0.8% muscle loss per year, contributing substantially to the estimated 1–2% yearly muscle loss from the age of 50 onwards [27].

A recent study of intensive care unit (ICU) patients on mechanical ventilation revealed that muscle mass loss occurred in a caudal-to-cranial direction over a period of 7 days. Notably, the lower limbs experienced the most significant loss within the initial 3 days, which then gradually decreased by day 7. In contrast, substantial muscle mass reduction in the upper limbs was observed after day 5, and abdominal muscle mass notably declined by day 7 [28].

## 4. Epidemiology

The prevalence of acute sarcopenia among inpatients is not well known. Diagnosing acute sarcopenia requires evidence of rapid declines in muscle mass and function, necessitating that these measurements are available from either before the illness or its early stages. Unfortunately, routine clinical practice does not typically include assessments of muscle mass and function, leading to frequent underdiagnosis of acute sarcopenia. An Italian study by Martone et al. showed that acute sarcopenia occurred in 14.7% of older patients in acute internal medicine and geriatrics care wards [29]. In rehabilitation settings, particularly among post-hip-fracture patients, the estimated prevalence of acute sarcopenia ranges from 28% to 69% [30]. After colorectal surgery, the prevalence of sarcopenia increased from 28.6% at baseline to 83.3% one week post-surgery [31]. Six months after gastrectomy for gastric cancer, the prevalence of acute sarcopenia was 20%, compared to a pre-surgery prevalence of chronic sarcopenia of 2% [32].

## 5. Pathophysiology

Muscle protein is constantly turned over with a net synthesis of approximately 1–2% per day in healthy adult humans, balanced by an equivalent rate of protein breakdown such that total muscle mass is maintained [33]. Sarcopenia results from an imbalance in these rates. The imbalance can arise from either a reduction in protein synthesis or an increase in protein breakdown that is greater than any change in the opposite direction and very small changes can lead to significant muscle loss over time [33,34]. Acute sarcopenia is considered to be caused by a combination of muscle disuse during bed rest [35,36], heightened inflammation [37], endocrinological stress response [38,39], and microRNAs involved in the regulation of muscle protein turnover [40,41,42].

### 5.1. Disuse

Research involving healthy volunteers has shown that bed rest leads to reductions in muscle quantity, strength, and aerobic performance [3,13,14,15,16,17,18,19,20,21,22]. Periods of disuse ranging from 10 to 42 days typically result in a muscle loss rate of approximately 0.5–0.6% of total muscle mass per day [35,43,44,45,46,47,48,49] with muscle strength declining variably between 0.3% [50,51] and 4.2% [49] per day.

The disproportionately greater loss of muscle strength compared to muscle mass is likely due to declines in neuromuscular recruitment and function associated with disuse [52].

Given that skeletal muscle mass turnover occurs at a relatively slow rate of about 1–2% per day, muscle atrophy due to disuse must be driven by a chronic, persistent disturbance in muscle protein balance. This means that, over an extended period, either muscle protein synthesis rates decrease, protein breakdown rates increase, or both processes occur simultaneously [15,21,30,31,32].

#### 5.1.1. Decreased Protein Synthesis

Extensive evidence indicates that immobilization impairs de novo muscle protein synthesis [35,44,53,54,55]. Muscle protein synthesis is regulated by growth factors, including insulin and IGF-1, which stimulate protein synthesis and increase muscle mass through the PI-3K-Akt-mTOR pathway [56]. Upon binding to IGF-1, the IGF-1 receptor (IGF-1R) phosphorylates an intracellular adaptor protein, insulin receptor substrate-1 (IRS-1), which in turn recruits and phosphorylates phosphoinositide 3-kinase (PI3K), followed by Akt phosphorylation. The PI3K/Akt pathway is crucial for myotube hypertrophy, and Akt activation in rat muscle prevents denervation-induced atrophy. Mammalian target of rapamycin (mTOR) is a downstream target of Akt. Immobilization negatively regulates the IGF-1/Akt/mTOR pathway, with the Akt-mTOR pathway being downregulated in rodent models of muscle atrophy [57]. It has been shown that IGF-1 levels, after initially dropping post-cardiac surgery, fail to return to normal in patients who develop post-surgery muscle wasting [42]. The decrease in muscle protein synthesis is an early phenomenon: in young men, a significant reduction in muscle phosphorylation status of Akt occurs after just 2 days of disuse [58]. Recent data also show that both Akt and p70S6K phosphorylation are suppressed between 1 and 4 days following the onset of disuse in younger adults [59] and in vivo muscle protein synthesis rates decline over a 24 h inactive period following surgery [60]. The reduction in Akt expression removes its inhibitory effect on catabolic pathways [56] and thus the reduction of muscle protein synthesis can also favor the increase in muscle protein breakdown. Animal models of disuse atrophy exhibit a marked increase in muscle protein breakdown rates following the onset of inactivity, along with a reduction in muscle protein synthesis rates [34,39,40,41,57,61,62].

#### 5.1.2. Increased Protein Breakdown

Protein breakdown in skeletal muscle occurs through several distinct processes, including caspase proteases involved in apoptosis [26], cathepsins integral to autophagy [63], the calcium-dependent calpain system [64], and the ubiquitin–proteasome pathway. Although the ubiquitin–proteasome pathway cannot degrade intact myofibrils without initial pre-processing by other pathways, it is considered the primary mediator of net skeletal muscle protein breakdown in humans [65,66,67]. During this process, specific proteins are marked for degradation through a three-step, enzymatic cascade [67]. This specificity is provided by a family of ubiquitin ligases, particularly the muscle-specific ligases muscle atrophy F-Box/atrogin-1 (MAFbx) and muscle-specific RING-finger protein 1 (MuRF1). These ligases are transcriptionally upregulated in various conditions that lead to muscle atrophy [67,68]. Notably, knocking out these specific ligases has been shown to induce partial resistance to muscle atrophy [67]. While it was once believed that MAFbx and MuRF1 operated together within a specific muscle “atrophy program” [68] recent research has revealed that they can also function independently during muscle atrophy [69]. Additionally, MuRF1 may play a role in inhibiting muscle protein synthesis pathways. Consequently, the transcriptional regulation of these ubiquitin ligases is widely regarded as a key indicator of increased ubiquitin–proteasome activity, protein breakdown, and muscle atrophy [65,66,67]. In the absence of direct, dynamic measurements of muscle protein breakdown rates, researchers often use the upregulation of the ubiquitin–proteasome system as an indicator of increased muscle protein degradation following disuse. For example, elevated mRNA levels of MAFbx [48] and/or MuRF1 [44,48,70], fork head box protein 01 (FOXO1; a known transcription factor for these ubiquitin ligases) [59], and the 20S proteasome 7 subunit [48] have been observed after 10–21 days of limb immobilization or bed rest in young individuals. Additionally, consistent with previous findings [70] recent research has shown an increase in polyubiquitinated proteins within skeletal muscle after prolonged bed rest [71]. Enhanced production of reactive oxygen species (ROS) during diseases and surgeries [72] can increase the activity of FOXO transcription factors, thereby elevating the expression of MuRF and Atrogin and promoting muscle catabolism.

Oxidative stress may also contribute to chronic low-grade inflammation [73]. Inflammatory signals further boost the expression of MuRF1 and Atrogin [56,74].

Muscle protein breakdown may indeed be elevated early during disuse, primarily mediated by the ubiquitin–proteasome pathway. Consistent with this, a previous study demonstrated that after 48 h of disuse in young men, there was an increase in the ubiquitination of high-molecular-weight proteins, alongside elevated mRNA levels of both MAFbx and MuRF1 [75]. Also, other studies have reported increased mRNA expression of MAFbx and/or MuRF1 following 2 [59,76], 3 [77], or 4 [59] days of limb immobilization in human volunteers. However, alterations in muscle protein breakdown do not appear to contribute to muscle loss during prolonged disuse in humans [78] as protein breakdown rates either remain unchanged or adaptively decrease [30,31]. Evidence indicates that short-term inactivity swiftly leads to a reduction in muscle protein synthesis rates and a concurrent, rapid, and potentially temporary increase in muscle protein breakdown. This combination of decreased synthesis and increased breakdown within skeletal muscles likely accounts for the significant muscle mass loss observed in the initial phase of inactivity. Subsequently, if inactivity persists, muscle protein breakdown rates return to near-normal levels. At this stage, gradual muscle loss continues at a slower pace, primarily due to a sustained decrease in both basal and post-prandial muscle protein synthesis rates.

### 5.2. Inflammation

Inflammation plays a pivotal role in the development of acute sarcopenia [79]. During acute inflammatory states, such as critical illness, infections, or trauma, proinflammatory cytokines like TNF-α, IL-6, and IL-1β are rapidly released. These cytokines activate catabolic pathways in muscle tissue, leading to increased proteolysis and muscle protein breakdown through the ubiquitin–proteasome system and autophagy–lysosome pathway. Additionally, inflammation induces oxidative stress, as reactive oxygen species (ROS) production is heightened, causing further damage to muscle cells and mitochondria. Damaged mitochondria release mitochondrial damage-associated molecular patterns (mDAMPs), which exacerbate inflammation by activating the NF-κB pathway and inflammasomes, such as NLRP3. This results in a vicious cycle of inflammation and muscle degradation [80,81,82,83]. The acute inflammatory environment also impairs anabolic signaling pathways and inhibits muscle protein synthesis, further contributing to rapid muscle atrophy. Inflammatory-induced mitochondrial dysfunction and impaired energy production hinder muscle repair and regeneration, accelerating the progression of acute sarcopenia. Experimental models, such as lipopolysaccharide-induced inflammation, have demonstrated that acute inflammatory signaling can swiftly lead to muscle catabolism, underscoring the critical link between inflammation and acute sarcopenia [84].

Notably, two recent meta-analyses have demonstrated a correlation between the inflammatory marker CRP and sarcopenia [85,86]. Fast-twitch fibers (type II) are particularly sensitive to the inflammatory processes associated with critical illnesses and are the most affected [87]. These fibers may undergo necrosis and be replaced by adipose tissue or fibrosis, which directly compromises daily living activities, especially those requiring power, in which these fibers are predominantly used [87,88,89].

#### GDF-15

Growth Differentiation Factor 15 (GDF-15), a member of the transforming growth factor-beta (TGF-β) superfamily, is significantly upregulated in various forms of stress. It has been demonstrated that patients who developed post-surgery muscle wasting after elective cardiac surgery failed to reduce the post-surgery increase in GDF-15 levels [42]. These findings were confirmed in patients post-aortic surgery [60]. In this study, pre-surgical levels of GDF-15 were higher in patients who subsequently lost more than 10% of the cross-sectional area of the rectus femoris muscle.

### 5.3. Hormonal Imbalances

Cortisol, a stress hormone, significantly increases during acute illness and stressful events [90]. It acts as a catabolic stimulus on muscles, and hypercortisolemia has been shown to exacerbate muscle mass and strength loss associated with bed rest. A controlled study demonstrated that timed hydrocortisone administration to healthy young men over a 28-day period of bed rest resulted in greater lean leg mass loss compared to a bed rest-only model [38]. A recent study demonstrated that the cortisol:cortisone ratio remained elevated after aortic surgery in patients who developed muscle wasting [91]. 11β-Hydroxysteroid dehydrogenase type 1 (11β-HSD1) regulates glucocorticoid exposure at the pre-receptor level. The type 1 isoform acts as an oxide reductase, converting cortisone to active cortisol. It can be induced by proinflammatory cytokines such as TNF-α. Recent studies have shown that skeletal muscle expression of 11β-HSD1 is negatively associated with grip strength in both men and women and with total lean mass in men [92]. Transgenic animal models have demonstrated that inactivation of 11β-HSD1 protects against skeletal muscle atrophy induced by exogenous glucocorticoids [93]. 11β-HSD1 has been identified as a major regulator of intramyocellular protein metabolism, influencing myotube size in both animal and human models and affecting the expression of various genes involved in protein synthesis, growth factors, and the ubiquitin–proteasome system [94].

### 5.4. MicroRNAs

MicroRNAs (miRNAs) are non-coding RNAs increasingly implicated in muscle wasting associated with various disease states. Several miRNAs interact with different pathways involved in muscle protein turnover. For instance, miR-29b inhibits IGF-1 and PI3K, leading to muscle atrophy [95]. Human muscle biopsies have shown that miRNAs such as miR-542 and miR-424 are overexpressed in individuals with intensive-care-unit-acquired weakness compared to controls, whereas miR-181a, miR-1, and miR-133b are underexpressed [40,41,42]. Furthermore, chronic obstructive pulmonary disease (COPD) has been linked to higher levels of miR-424 and miR-542, suggesting that the underlying disease state may stimulate their expression [40,41].

## 6. Assessment

Acute sarcopenia is characterized by a significant decrease in muscle strength and mass and performance compared to baseline levels, meeting the established cut-offs defined for the diagnosis of chronic sarcopenia (Table 1) [2,18,19].

### 6.1. Muscle Mass Measurement

Computed tomography (CT) and magnetic resonance imaging (MRI) are considered the gold standards for non-invasive muscle mass assessment [96] and dual energy X-ray absorptiometry (DEXA) is the most commonly used technique for evaluating body composition [97]. However, these methods are not suitable for assessing acute sarcopenia due to concerns about radiation exposure (for CT and DEXA) [96] and long duration of the procedure and high costs (for MRI). Bioelectrical impedance analysis (BIA) is a non-invasive method that indirectly evaluates body composition by assessing tissue conductivity. Small electrical currents pass through intra- and extracellular fluids, varying with tissue characteristics. Skeletal muscles, having the largest volume and lowest resistance among body tissues, allow most of the BIA current to flow through them. In contrast, adipose tissue displays current resistance [98]. Validated conversion equations are then used to estimate muscle and fat mass, with reference values established for different ethnicities and age groups [99,100,101,102]. Notably, the Sergi equation is based on older European populations [100]. BIA offers several advantages: it is inexpensive, easily reproducible, and suitable for repeated measurements in both ambulatory and bedridden patients. Additionally, BIA results under standard conditions correlate well with MRI findings. Muscle quantity measured by BIA also shows a good correlation with muscle echography [103]. However, BIA’s accuracy can be significantly affected by fluid balance [104,105]. Moreover, it is currently not recommended for use in individuals with implantable cardiac devices, although emerging research suggests it may be safe in these cases [106]. Ultrasound is an emerging technique that is gaining increasing attention for evaluating muscle quantity and quality. It can detect changes in muscle thickness and cross-sectional area even over short periods [107]. Measurements of bilateral anterior thigh thickness (BATT) using ultrasound show excellent consistency between different observers and the same observer over time [108]. The EWGSOP2 endorses ultrasound for clinical sarcopenia assessment, [2] and a consensus protocol has been established [107,109]. The threshold values for reduced muscle mass using BATT ultrasound are 3.86 cm for women and 5.44 cm for men [108].

The Sarcopenia Definition and Outcomes Consortium (SDOC) criteria suggest that lean mass measured by DXA should be excluded from the definition of sarcopenia because it has shown to be a weak predictor of negative health outcomes [19]. Given that the reduction of muscle mass has historically been considered a key aspect of sarcopenia, the SDOC allows for the potential inclusion of more precise measures of muscle mass in the future, provided these measures are shown to be linked to adverse clinical outcomes.

### 6.2. Muscle Quality Measurement

The assessment of muscle quality has become increasingly important as it appears to decline before muscle mass does. Additionally, muscle quality is independently linked to significant health outcomes [110]. It is affected by the proportion of non-contractile and contractile elements within the skeletal muscle [111] and can be histologically characterized by fat infiltration and fibrosis [1,112,113]. Activation of genes promoting fibrosis has been observed in both critical illness myopathy and in intensive care unit patients infected with SARS-CoV-2 [114]. Muscle echography is capable of assessing muscle quality through the determination of muscle echointensity (EI) [115,116] and muscle stiffness (SI) [117]. EI, which indicates the brightness of an ultrasound image, is expressed as a value between 0 (black) and 255 (white) arbitrary units (AU) in a region of interest. Fat and fibrous tissues appear whiter than muscle fibers. SI, calculated with specialized software, inversely correlates with muscle quality, meaning higher stiffness indicates poorer muscle quality. There is currently no consensus on which parameter is preferable for evaluating muscle quality via ultrasound. Increased echogenicity of the rectus femoris has been observed in cases of acute sarcopenia among COVID-19 patients [118] and COVID-19 survivors [111,119].

### 6.3. Muscle Strength Assessment

Muscle strength can be assessed using either grip strength or the chair stand test [2]. Grip strength moderately correlates with strength in other parts of the body. Due to its simplicity, it is recommended for the assessment of acute sarcopenia in hospital settings. The handgrip test is performed using a calibrated handheld dynamometer. Individuals are positioned with their elbows bent at a 90° angle and forearms in a neutral position, and they are instructed to “squeeze as hard as they can”. The Jamar dynamometer is a validated and widely used tool for measuring grip strength, although other brands are also being considered. The cut-off values for low grip strength are less than 27 kg for men and less than 16 kg for women [2] for the EWGSOP2, less than 28 kg in men and less than 18 kg in women for the AWGS [18], and less than 35.5 kg in men and less than 20 kg in women for the SDOC criteria [19]. The chair stand test can serve as an indicator of leg muscle strength. It measures the time it takes for an individual to stand up from a seated position five times without using the arms [120]. A duration of more than 15 s to complete this task indicates low muscle strength [2].

### 6.4. Muscle Performance Assessment

Muscle performance is typically assessed using the Short Physical Performance Battery (SPPB) or measuring gait speed [2]. The SPPB comprises three subtests that evaluate standing balance, usual gait speed over a short distance, and the ability to rise from a chair. For the standing balance test, the subject is asked to stand in three progressively challenging positions for 10 s each: a side-by-side feet position, a semi-tandem position, and a full tandem position. In the gait speed subtest, the subject walks at usual pace over a 4 m course, starting from a stationary position. The faster of the two trials (measured in seconds) is used to calculate the summary score. For the chair stand subtest, the subject is asked to rise from and sit back in a chair five times as quickly as possible with their hands folded across the chest. The performance is recorded as the total time (in seconds) taken to complete the task. The results of these three timed tasks are scored from 0 (worst performance) to 4 (best performance) based on predetermined cut-off points. The sum of the scores from the three subtests produces a summary score of physical performance, ranging from 0 (worst performance) to 12 (best performance) [121]. An SPPB score of ≤8 indicates reduced physical performance according to the EWGSOP2 criteria [2]. Considering that the Asian population is usually more active the cut-off point for the SPPB according to AWGS is ≤9 [18].

Alternatively, physical performance can be measured using gait speed. A gait speed of ≤0.8 m/s (over a 4-meter walk) indicates a reduction in muscle performance [2] according to the EWGSOP2 criteria, whereas the cut-off is ≤1 m/s (over a 6 m walk) for the AWGS [18] and <0.8 m/s for usual gait speed for the SDOC criteria [19].

## 7. Biomarkers of Acute Sarcopenia

Inflammatory biomarkers such as C-reactive protein (CRP), granulocyte–monocyte colony-stimulating factor (GM-CSF), interferon-γ (IFNγ), interleukins (IL-6 and IL-8), myeloperoxidase (MPO), P-selectin, and tumor necrosis factor-α (TNF-α) may naturally be elevated in cases of acute sarcopenia. However, distinguishing fluctuations in these biomarkers specifically related to acute changes in muscle mass and function from those driven by the underlying acute condition that triggered sarcopenia presents a significant challenge. The complex interplay between the inflammatory response and muscle degradation makes it difficult to clearly differentiate these influences.

Possible innovative biomarkers of acute sarcopenia include a range of molecules that reflect the underlying mechanisms of rapid muscle loss (Table 2). Growth Differentiation Factor 15 (GDF-15) is a key biomarker, as it is associated with inflammation and muscle wasting [42,60]. MicroRNAs (miRNAs) are also significant; specific miRNAs such as miR-29b [122], which inhibits IGF-1 and PI3K pathways, contribute to muscle atrophy. Elevated levels of miR-542 and miR-424, and decreased levels of miR-181a, miR-1, and miR-133b, have been observed in acute muscle weakness [40,41,42]. A promising but not yet demonstrated biomarker of acute sarcopenia might be myokine fibroblast growth factor 21 (FGF21). Muscle-derived FGF21 is produced in large quantities in response to muscular stress, such as mitochondrial dysfunction, and respiratory chain blockage. FGF21 reduces oxidative stress damage to skeletal muscle mitochondria, helps prevent muscle myopathy, and maintains muscle metabolic balance. Notably, FGF21 production is sensitive to protein content, increasing when protein intake is low. Higher circulating FGF21 levels have already been linked to chronic sarcopenia in older people but its role as a biomarker of acute sarcopenia remains to be explored [123,124,125].

## 8. Risk Factors

During acute illness, the upregulation of catabolic pathways generates energy to combat the disease. However, this process may lead to a loss of muscle mass and function as a trade-off. Identifying risk factors for acute sarcopenia is crucial for recognizing patients at risk and preventing both the immediate and long-term consequences of the condition. Some risk factors are modifiable, while others are not. Nevertheless, early detection of these factors can facilitate the implementation of countermeasures to prevent or mitigate the severity of acute sarcopenia.

### 8.1. Old Age

Advanced age is a significant risk factor for the development of acute sarcopenia. Multiple underlying mechanisms in older adults contribute to the onset of this condition.

#### 8.1.1. Inflammaging

As people age, systemic basal inflammatory mediators increase independently of acute immune challenges, a phenomenon known as inflammaging [126]. Chronic inflammation is believed to be a major factor contributing to immunosenescence [127] and predisposes older people to various diseases with a shared inflammatory origin, termed the “diseasome of inflammaging” [128]. Inflammation, by activating catabolic pathways, also plays a role in the development of acute sarcopenia. The presence of inflammatory cells [129] and cytokines [130] has been observed in muscle biopsies of individuals with intensive-care-unit-acquired weakness (ICUAW), a condition marked by acute loss of muscle mass and function, which is closely related to acute sarcopenia.

#### 8.1.2. Immunosenescence

Immunosenescence is marked by a reduced capacity to respond to new antigens [131] and inability to deactivate inflammatory signals once an infection has been resolved. This results in persistently high levels of inflammatory markers, which trigger catabolic signals in muscles, promoting the development of sarcopenia. Additionally, aging alters neutrophil migratory dynamics, leading to increased tissue damage due to excessive release of neutrophil elastase, further contributing to muscle loss [132].

#### 8.1.3. Mitochondrial Damage

Aging is marked by increased generation of reactive oxygen species (ROS) in muscle mitochondria, particularly those under the sarcolemma [104,105,106], along with mitochondrial vacuolization and enlargement. Additionally, there is an increase in damaged mitochondrial DNA (mtDNA), the release of apoptogenic factors usually stored in the mitochondrial intermembrane [133,134], and elevated levels of mitophagy, while mitochondrial biogenesis declines. These age-related mitochondrial changes contribute to the susceptibility to developing sarcopenia [135,136,137]. One of the primary mechanisms regulating mitochondrial quality and turnover is the mTORc1 pathway. Its inhibition leads to decreased mitochondrial biogenesis, reduced mtDNA production, increased mitochondrial damage, and impaired mitochondrial quality [135]. Inflammation, physical inactivity, increased adiposity, and type 2 diabetes mellitus all interact with the mTORc1 pathway, resulting in decreased muscle protein synthesis.

#### 8.1.4. Gut Microbiota

As individuals age, the gut’s ability to contain microbes and their byproducts diminishes [138]. These harmful microbial products can then leak into surrounding tissues and the bloodstream, exacerbating the existing inflammaging [126]. Additionally, since the gut microbiota influences the immune system’s memory mechanisms [139], age-related changes in its composition [140] can worsen immunosenescence-related muscle damage.

#### 8.1.5. Oral Health and Protein Intake

Anorexia of aging [141,142], along with poor dental health, reduced salivation, and decreased strength in the masticatory muscles and tongue [143] can lead to swallowing difficulties, reducing the intake of protein necessary for muscle anabolism. Consequently, these changes can promote chronic sarcopenia that favors the development of acute sarcopenia when an acute trigger occurs.

#### 8.1.6. Anabolic Resistance

Anabolic resistance refers to the decreased ability of muscles in older adults to synthesize protein in response to anabolic stimuli, such as protein consumption and physical activity. This decline in muscle response makes it harder for older individuals to build or maintain muscle mass. Anabolic resistance is driven by several mechanisms: increased splanchnic sequestration of amino acids, reduced post-prandial muscle perfusion, decreased muscle uptake of dietary amino acids, impaired anabolic signaling for protein synthesis, compromised digestive capacity [144,145,146,147,148], and reduced nutrient delivery due to macrovascular and microvascular changes [149].

Anabolic resistance can also be triggered by prolonged disuse [150]. For example, seven days of bed rest can cause approximately a 35% decline in the muscle protein synthetic response to essential amino acid ingestion in older individuals [55,151]. Extended periods of immobilization (i.e., 14 days) can induce anabolic resistance in response to hyperaminoacidemia even in young men [151,152]. Anabolic resistance likely worsens the catabolic state observed in critically ill patients, suggesting that traditional protein goals should be increased to compensate for this effect [153].

#### 8.1.7. Endocrine Dysregulation

Age-related changes in endocrine signals contribute to increased catabolic and decreased anabolic pathways in muscles. Cortisol levels slightly rise with age [154], leading to muscular weakness [155]. Additionally, serum levels of the androgen precursor dehydroepiandrosterone sulfate (DHEAS) decline, resulting in an increased cortisol:DHEAS ratio and a state of relative cortisol excess. Acute illness or stress exacerbates this imbalance, further compromising muscle health [156]. Anabolic hormones such as testosterone, growth hormone (GH), and insulin-like growth factor 1 (IGF-1) decrease after the age of 60, contributing to muscle loss. Testosterone inhibits the production of myostatin and reactive oxygen species (ROS), prevents apoptosis, enhances myosatellite stem cells, accelerates muscle IGF-1 expression, regulates skeletal muscle metabolism, and increases muscle protein synthesis and mass in elderly men. Low GH/IGF-1 expression decreases protein anabolism in skeletal muscle cells, leading to structural and functional changes that favor the development of sarcopenia [157].

### 8.2. Inactivty

Inactivity is associated with significant metabolic disruptions [158]. Studies on step reduction in both younger (mean age 29 years) and older (mean age 69–72 years) healthy adults have shown a decrease in insulin sensitivity [159,160]. Even after returning to previous activity levels for 14 days, changes in glucose tolerance and proinflammatory cytokines were not fully reversed [158,161]. Chronic muscle disuse also leads to excessive production of reactive oxygen species, causing additional mitochondrial damage [153]

#### Immobility

Disuse leads to rapid skeletal muscle loss [53,162] through several mechanisms, including inflammation, myostatin, Atrogin-1/Muscle atrophy F-Box (MaFbx)/Muscle ring finger 1 (MuRF1), and IGF-1-AKT-mTOR pathways [26,163,164]. The mechanisms responsible for early muscle loss during disuse differ from those involved in prolonged disuse. In the initial phase of disuse (10 to 42 days), the rate of muscle loss is higher, approximately 0.5–0.6% per day [165,166]. In contrast, during extended periods of disuse (17 weeks), the rate of muscle loss decreases to about 0.1% per day [50]. Interestingly, immobilization leads to changes in muscle composition similar to those seen in chronic sarcopenia [167,168]. For instance, 14 days of bed rest in middle-aged individuals primarily resulted in a reduction of type 2a muscle fibers and satellite cell content [169]. The negative effects of bed rest on muscles are evident even in healthy individuals [20,35] but are more pronounced in older adults [26]. For example, just five days of bed rest in older adults can lead to a reduction in lean leg mass [36]. Martone et al. demonstrated that patients who developed acute sarcopenia during hospitalization in internal medicine and geriatric wards spent more time in bed compared to those who did not develop acute sarcopenia (5.1 days vs. 3.2 days) [29]. These findings were corroborated in intensive care units, where longer ICU stays were associated with increased loss of quadriceps muscle mass [170]. Lastly, it is crucial to recognize that extended bed rest can impair baroreceptor reflexes, leading to orthostatic hypotension. This condition may cause a fear of falling, further reducing physical activity and worsening sarcopenia [171,172].

### 8.3. Malnutrition

A reduced intake of essential nutrients such as protein, vitamin D, and calcium impairs the maintenance of muscle mass, strength, and performance [2,158,173]. Specifically, a decreased intake of foods rich in essential amino acids, particularly leucine, may result in fewer physiological stimuli for muscle mass growth. Leucine is known to activate the mTORc1 pathway, enhancing mitochondrial biogenesis and muscle growth [174].

#### 8.3.1. Anorexia

During acute illnesses, decreased appetite is a major contributor to malnutrition [142,175,176], leading to insufficient protein intake and promoting the development of acute sarcopenia. The prevalence of decreased appetite is reported to be 64% during hospitalization and 28% after discharge [177,178,179]. A diminished appetite at the time of hospital admission is linked to several adverse outcomes, including reduced muscle strength during and after hospitalization, impaired mobility skills during hospitalization, and decreased physical performance both during and after the hospital stay [142,175,176,180].

#### 8.3.2. Obesity

The relationship between obesity and sarcopenia is bidirectional. On one side, the lower basal metabolic rate of aged fat-free mass promotes the accumulation of fat mass. On the other side, obese individuals tend to be less active than their lean counterparts, further increasing fat accumulation. Obesity can create resistance to anabolic stimuli, such as growth factors, hormones, amino acids, and exercise, which impairs muscle mass anabolism. Moreover, obesity leads to systemic low-grade inflammation, especially from visceral fat, which secretes various proinflammatory cytokines like IL-6 and TNF-α. This inflammation is also a predisposing factor for sarcopenia though the activation of catabolic pathways [181].

The coexistence of obesity and sarcopenia configures a condition named sarcopenic obesity (SO) [182]. SO should be recognized as a distinct clinical condition, separate from either obesity or sarcopenia alone. This is due to two key factors: the abovementioned bidirectional, pathological interaction between the accumulation of body fat and the loss of skeletal muscle mass and function and the combined negative effects of obesity and sarcopenia, which together create a significantly higher risk of metabolic diseases and functional impairments than the risks posed by each condition individually [183,184,185,186,187,188]. This synergy increases the overall health burden beyond what would be expected from simply adding the risks of obesity and sarcopenia together. Individuals with sarcopenic obesity are likely at an elevated risk of developing acute sarcopenia compared to those affected by either obesity or sarcopenia alone. However, further research is needed to confirm this hypothesis and fully understand the underlying mechanisms contributing to this increased vulnerability.

### 8.4. Hospitalization and Delirium

Hospitalization in both medical and surgical units has been linked to the onset of acute sarcopenia [189]. Muscle mass loss is a well-documented outcome of critical illness, and a condition closely related to acute sarcopenia—intensive-care-unit-acquired weakness (ICUAW)—has been observed in patients in intensive care units [164]. Hospitalization is often associated with prolonged bed rest, with up to 50% of hospitalized older adults being active for only about 30 min per day [190]. Furthermore, the inflammatory response can trigger catabolism, leading to reductions in muscle mass and function. Conditions with a strong inflammatory component, such as sepsis, are associated with significant muscle wasting, resulting in the loss of more than 10% of lower limb muscle mass in less than two weeks [191,192]. These effects are further exacerbated by aging [35,36]. Notably, hospitalization can be complicated by delirium. In its hypoactive form, delirium directly reduces physical activity. In its hyperactive form, it may necessitate antipsychotic treatment, which also tends to increase bed rest. Additionally, some patients may develop pressure ulcers despite preventive measures, adding to the catabolic burden and further limiting their voluntary movements [131]. Older adults who are dependent at home face a higher risk of worsening sarcopenia during and after hospitalization [193].

### 8.5. Chronic Sarcopenia

Individuals with chronic sarcopenia, characterized by a long-term reduction in muscle mass and function, are at a heightened risk of further muscle decline when faced with acute triggers [164]. Multimorbid individuals are at a higher risk of developing sarcopenia [194], as they tend to be less active than those without multiple chronic conditions and exhibit increased inflammation due to their disease burden. Additionally, sarcopenic individuals, especially the comorbid ones, are more likely to be hospitalized compared to those without sarcopenia, increasing their likelihood of encountering acute triggers for acute sarcopenia [195]. Furthermore, sarcopenic patients tend to have longer hospital stays, making them more susceptible to the adverse effects of immobilization during acute illness [196].

### 8.6. Drugs

Corticosteroids are known to have catabolic effects on muscles (e.g., prednisone, dexamethasone, methylprednisolone, hydrocortisone). It has been demonstrated that healthy young individuals on bed rest lose more muscle when given hydrocortisone compared to bed rest alone [38].

Numerous other medications can also cause weight and muscle mass loss due to various factors such as anorexia (caused by opiates, antibiotics, non-steroidal anti-inflammatory drugs, digoxin, metformin, anticholinergics, iron, potassium), malabsorption (caused by metformin, proton pump inhibitors, antacids, antibiotics), muscle disuse (caused by sedatives, neuroleptics), motor neuron loss, and endocrine dysfunction (caused by hormonal therapy in cancer, glucocorticoids) [197,198].

Non-steroidal anti-inflammatory drugs (NSAIDs) can sometimes lead to gastropathy, which may limit calorie and protein intake, ultimately affecting muscle mass. However, research by Trappe et al. [199] demonstrated that older adults who took cyclooxygenase (COX) inhibitors daily during a 12-week resistance training program saw 25–50% greater improvements in muscle mass and strength compared to a placebo group following the same regimen. This indicates that COX pathways are involved in controlling muscle protein turnover and mass and it is possible that COX inhibition has a more pronounced effect in slowing muscle protein breakdown than on protein synthesis itself. Thus, NSAIDs might also prevent muscle loss with age.

### 8.7. Surgery

Surgery can contribute to muscle wasting through several mechanisms, including inflammation, oxidative stress, immobilization, and reduced oral intake. The trauma associated with surgery triggers a significant inflammatory response, which has long been considered a primary factor in post-surgical muscle wasting [200]. Another study examining the metabolic impacts of surgery found that the circulating levels of chemokines C-C motif chemokine 23 (CCL23) and IL-8 were positively correlated with the levels of circulating amino acids, whereas IL-5 was negatively correlated with amino acid levels post-surgery [91]. These findings suggest that various inflammatory factors play a role in the reduction of muscle mass following surgery. Oxidative stress during surgery can arise from several sources. Ischemia–reperfusion injury, in particular, is known to induce oxidative stress in various surgical contexts, such as transplantation, aortic unclamping, limb tourniquet release during orthopedic procedures, and reperfusion during coronary bypass surgery [201]. Moreover, emergency surgery is associated with greater oxidative stress than elective surgery [202]. Pre-operatively, levels of miR-424 and miR-542-3p in muscle biopsies were found to be higher in patients undergoing aortic surgery who went on to lose muscle [40,41]. Additionally, emergency surgeries are linked to higher levels of oxidative stress compared to elective surgeries [203]. miR-424 and miR-542 play a direct role in regulating muscle protein homeostasis. Both miR-424-5p and miR-542-3p target the protein synthesis machinery, with miR-542-3p also contributing to mitochondrial dysfunction and promoting TGF-β signaling [40]. Conversely, miR-422a, which is negatively associated with muscle loss following surgery, inhibits TGF-β signaling by targeting SMAD4 [203]. Surgery poses a high risk of immobility due to various factors, including pain, the necessity for non-weight-bearing status following certain procedures, and a reduced functional state resulting from underlying disease or the effects of surgery and anesthesia. These factors collectively contribute to development of acute sarcopenia. Protein intake is crucial for muscle maintenance, and deficiencies due to either reduced intake or malabsorptive states can directly lead to muscle wasting [1]. This issue is particularly relevant for surgical patients. For instance, those with an “acute abdomen” might experience anorexia or vomiting for several days before an emergency laparotomy. Additionally, most surgeries require pre-operative fasting to reduce the risk of aspiration [204]. Furthermore, patients undergoing certain procedures, especially gastrointestinal surgeries, are often advised to continue fasting post-operatively for various reasons, such as minimizing the risk of vomiting and protecting surgical anastomoses. Even when feeding resumes, the gradual return to a normal diet is typically prolonged [205].

After hip replacement surgery, reductions in muscle cross-sectional area have been observed [46,206], persisting for up to 3.5 years post-surgery [207]. Similarly, a decrease in psoas muscle area within the first year following endovascular aortic aneurysm repair has been reported [67], along with a reduction in fat-free muscle mass after cardiac bypass surgery [208]. Additionally, research involving older adults admitted electively for colorectal surgery has shown acute declines in handgrip strength and muscle quantity, as measured by BIA [209] and BATT [31].

### 8.8. Stroke

Stroke patients are at a higher risk of developing severe sarcopenia due to various factors such as muscle atrophy from paralysis and disuse, spasticity, inflammation, denervation and reinnervation, and impaired feeding and intestinal absorption [210,211]. Neuropathy can start as soon as four hours after the onset of a stroke [212] and transsynaptic inhibition of spinal alpha motor neurons results in a reduction of motor units [212]. Additionally, patients with acute stroke generally experience significant inactivity, engaging in less than 40 min of physical activity during hospitalization [213]. Sarcopenia has a detrimental impact on clinical outcomes in stroke patients [214], as it hinders functional recovery and delays the return to home [215]. The prevalence of acute sarcopenia in stroke patients is estimated to range from 8.5% to 33.8% [214,216,217,218,219], and it tends to increase progressively in the days following the acute event. Within the first 10 days post-stroke, the prevalence is about 29.5%, rising to 51% between 10 days and 1 month after the stroke [220].

### 8.9. COVID-19

Coronavirus disease (COVID-19) can lead to the development of acute sarcopenia through several mechanisms: inflammation, increased ROS generation, immobility and reduced physical activity, anorexia, hospital-related malnutrition and subsequent weight loss, direct cytopathic effects of SARS-CoV-2 on muscles, hypoxia, and steroid therapy [221]. The acute inflammatory response to the infection has a high potential to damage a wide range of organs, including muscles [131,222]. Additionally, it raises caloric demand, which is often inadequately met. COVID-19-related mitochondrial damage has a significant potential to induce sarcopenia. Ferritin, an acute-phase reactant and key regulator of iron homeostasis, can directly affect mitochondrial energy production. This interaction increases ROS generation and heightens cellular susceptibility to damage and cell death [223,224]. Mitochondrial impairment, in combination with accumulation of amyloid and muscle shift to fast fatigable fibers, seem to be involved in the persistent reduction of exercise capacity after the resolution of SARS-CoV-2 infection [225]. During the acute stage of COVID-19, anosmia (experienced by 41.0–52.7% of cases) and ageusia (experienced by 38.2–43.9% of cases) [226,227,228] may lead to a reduction in oral intake. Additionally, within a month of infection, up to 30% of individuals with olfactory dysfunction and 20% with gustatory dysfunction may not recover, and those who do may experience paraosmias or distorted taste, potentially affecting oral intake even in COVID-19 survivors [229]. Furthermore, symptoms such as nausea and diarrhea, along with liver dysfunction commonly seen in some patients, may exacerbate anorexia and impair nutrient assimilation [230]. Inadequate nutrition in COVID-19 patients and survivors can also contribute to the development of sarcopenia. Data from an international survey revealed that unhealthy food consumption increased during the pandemic [231], alongside a reduction in vegetable intake [232]. Additionally, there was a rise in compulsive eating habits [233,234]. These dietary changes can significantly contribute to the onset of sarcopenia through various mechanisms. Clinical observations indicate that during the acute phase of infection, patients were at risk of losing 5–10% of their body weight [235,236]. During the COVID-19 pandemic, reduced physical activity or bed rest was linked not only to the acute disease but also to widespread lockdown measures and social distancing [231,233]. Extended hospitalization periods could lead to greater muscle damage, potentially in an exponential rather than linear fashion. Immobilization during COVID-19 hospitalization differs significantly from immobilization due to other conditions. COVID-19 patients often experience profound weakness, spending extended hours on high-flow oxygen therapy or in the prone position. According to Mayer et al., ICU stays were associated with a median decrease of 18.5% in rectus femoris muscle mass between the first and seventh day [237]. Sarcopenic dysphagia, which can acutely develop in COVID-19 patients, may create a self-perpetuating cycle of events [238]. Coupled with the proanorectic effects of inflammation and hypoxia, this condition can significantly reduce food intake [239]. Given that skeletal muscles express ACE2 abundantly, it is possible that SARS-CoV-2 exerts a direct cytopathic effect on these muscles [240].

Hypoxia can contribute to acute sarcopenia by negatively affecting various functions. It diminishes the sensation of hunger by stimulating leptin production [223,241]. Hypoxia is also associated with higher levels of myostatin and a shift from the IGF-1/Akt pathway to the IGF-1/ERK pathway, which stimulates myogenesis but not differentiation in skeletal muscle [239]. Sarcopenic involvement of the diaphragm and intercostal muscles impairs ventilatory function, further worsening hypoxia and perpetuating the vicious cycle [241]. Parenteral steroids have been administered to patients with severe COVID-19 and respiratory failure requiring oxygen therapy. Steroids are known to increase protein turnover in skeletal muscles, resulting in decreased muscle mass and outright wasting [242,243]. Post-COVID-19 emotional disorders have been observed [244,245], leading to an increase in individuals experiencing depressed mood. This condition can result in abulia, negatively impacting physical activity [246].

**Figure 3:**
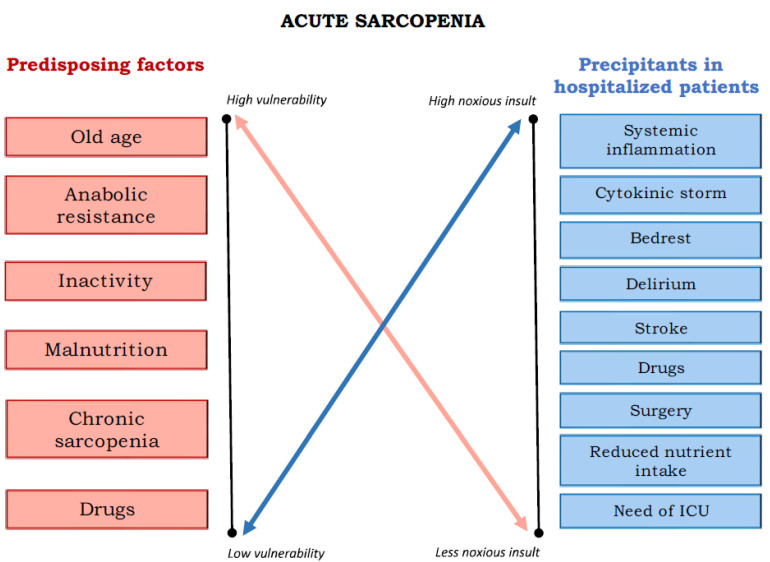
Predisposing and precipitating risk factors for acute sarcopenia.

In summary, similar to many geriatric syndromes, individuals with higher baseline vulnerability require lower levels of acute stress to develop acute sarcopenia. Conversely, in healthy young adults, a much more intense stress is needed to induce acute sarcopenia (Figure 3).

Figure 3 outlines the predisposing factors and precipitating events that contribute to the development of acute sarcopenia, particularly in hospitalized patients. Predisposing factors include old age, anabolic resistance, inactivity, malnutrition, chronic sarcopenia, and chronic drug use, which increase vulnerability to muscle loss. Factors such as systemic inflammation, cytokine storm, bed rest, delirium, stroke, acute drug side effects, surgery, reduced nutrient intake, and intensive care unit (ICU) stay represent highly noxious insults that can precipitate acute muscle loss. The figure emphasizes the interaction between baseline vulnerability and acute health events, leading to rapid muscle degradation. Individuals with higher baseline vulnerability require lower levels of acute stress to develop acute sarcopenia. Conversely, in healthy young adults, a much more intense stress is needed to induce acute sarcopenia.

## 9. Consequences

The implications of muscle mass in various clinical scenarios have reshaped our understanding of muscle. It is now recognized as a crucial component of metabolism with endocrine and paracrine functions, rather than merely a mechanical organ [247]. Indeed, muscles significantly influence physical recovery, rehabilitation potential, and long-term function following hospitalization. Acute sarcopenia can lead to chronic sarcopenia, as recovery from acute muscle wasting is often incomplete, particularly in older adults [164,248,249,250]. People with chronic sarcopenia are less active and display an increased risk of gaining weight and obesity. Obesity and sarcopenia create a vicious cycle where muscle loss leads to decreased physical activity and metabolic rate, promoting fat gain. Excess fat causes chronic inflammation and metabolic dysregulation, further accelerating muscle degradation. This results in reduced mobility, increased fat deposition, and insulin resistance, exacerbating both conditions. Acute sarcopenia is linked to higher financial costs due to longer hospital stays, increased ICU admission risk [251], transplantation graft failure [252], greater rehabilitation needs, higher rates of discharge to institutional care, and increased social care requirements [164,253,254,255,256]. Moreover, acute sarcopenia increases the risk of falls both during and after hospital stay and consequently the risk of fractures. In addition, acute sarcopenia in the oral district can cause dysphagia leading to ab ingestis pneumonia which further increases the length of hospital stay [164,173]. Acute sarcopenia has been linked to higher mortality rates in older patients hospitalized in medium-complexity units [256], in COVID-19 patients [251], and in those undergoing liver transplantation [252,257], colorectal cancer surgery across all age groups [258,259], and endovascular aortic aneurysm repair [260]. Interestingly, in colorectal cancer patients, mortality was highest among those with acute-on-chronic sarcopenia compared to those with either acute sarcopenia or chronic sarcopenia alone (with the lowest mortality observed in the chronic sarcopenia group) [258]. This indicates that acute muscle wasting plays a more significant role in determining mortality than baseline sarcopenia status.

**Figure 4:**
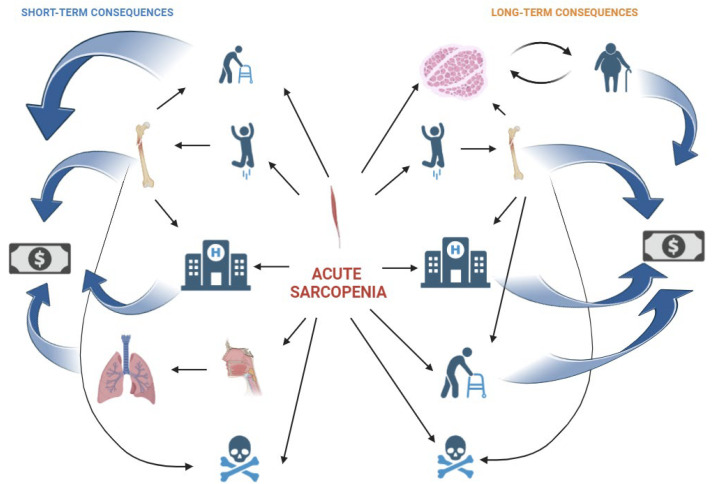

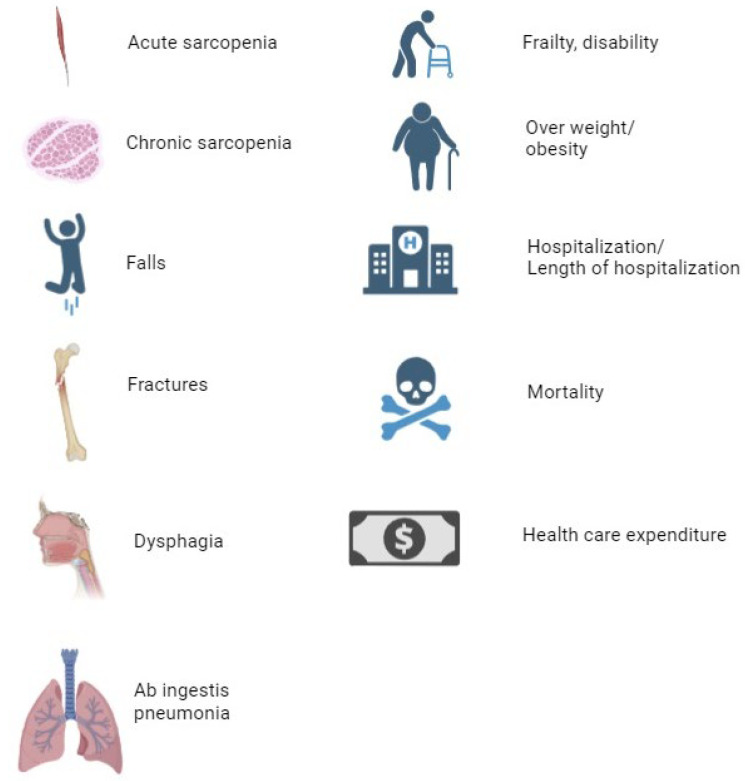
Consequences of acute sarcopenia.

Figure 4 illustrates the short-term and long-term consequences of acute sarcopenia, highlighting its impact on overall health and healthcare systems. Short-term consequences include increased risk of falls, fractures, prolonged hospital stay, respiratory complications, acute frailty/disability, dysphagia due to acute sarcopenia in muscle of the oral district and consequent risk of ab ingestis pneumonia, and increased healthcare costs and risk of death. Long-term consequences include chronic sarcopenia reducing physical activity and increasing the risk of becoming overweight or obese. Moreover, there is a long-term risk of falls and fractures, disability, reduced quality of life, increased risk of hospital readmissions, and higher mortality rates. The figure also depicts the economic burden associated with long-term care needs and increased healthcare utilization. These consequences underscore the need for early intervention to prevent and manage acute sarcopenia.

## 10. Interventions

The efficacy of chronic sarcopenia interventions in treating acute sarcopenia remains uncertain due to differing underlying mechanisms. Acute sarcopenia is characterized by heightened systemic inflammation and immune–endocrine dysregulation. Inflammation, whether acute or chronic, can impede the body’s response to exercise or protein intake, leading to anabolic resistance [261]. Acute sarcopenia progresses rapidly, potentially rendering traditional treatments insufficiently fast-acting [164]. Furthermore, implementing community-based interventions in a hospital setting may prove impractical. Various interventions have been safely tested for the treatment of acute sarcopenia in diverse populations and settings. However, treatment approaches may need to be tailored to individual needs.

### 10.1. Physical Activity

The potential for exercise to reverse acute sarcopenia appears encouraging but remains unconfirmed [262].

Minimizing bed rest during hospitalization is highly recommended [263] due to its broad health benefits beyond improving muscle function [164]. A variety of physical activity interventions have been tested for treating hospitalized patients. These include strength and balance training [264,265,266,267], early and/or increased mobilization [268,269,270,271,272,273], group exercise [274], water exercise/physiotherapy [275], chair-based exercise [265,276], seated side-tapping [277], pedal exercisers [278,279], and progressive weight-bearing exercise in orthopedic rehabilitation [280,281,282] using specialized harnesses where appropriate. Exercise training activates the mechanistic target of rapamycin (mTOR) pathway and insulin-like growth factor 1 (IGF-1), promoting muscle protein synthesis [283]. Resistance exercise is the most effective approach, backed by substantial evidence [284], for treating chronic sarcopenia [285] as it is able to improve muscle strength and physical performance if performed for three to eighteen months. Indeed, it specifically enhances satellite cell recruitment and muscle hypertrophy [286]. Thus, resistance exercise seems to be a promising option for the treatment of acute sarcopenia. Previous studies showed that hospitalized patients who performed resistance exercise improved their muscle strength, power, and performance compared to individuals treated with usual care [287]. Three randomized controlled trials (RCTs) implemented progressive resistance exercise (RE) using specialized equipment such as machines, external weights, or a cycle ergometer, while four other RCTs focused on bodyweight-based resistance exercises. On average, the RE sessions lasted between 20 to 40 min and were conducted 5 to 7 consecutive days each week. Furthermore, in five of the RCTs, participants performed the RE exercises more than once daily, with sessions occurring up to twice a day [287].

However, none of the trials included in the review specifically selected participants with sarcopenia, nor did they evaluate sarcopenia levels before and after the treatment [287].

Also, a comprehensive physical training program, incorporating resistance exercises with machines and/or weights along with gait and balance training, significantly enhanced physical performance (such as gait speed and SPPB) and muscle strength [288,289]. In this study the multicomponent intervention consisted of two daily sessions performed in the morning and in the evening. Each session lasted 20 min and they were conducted over 5 to 7 consecutive days, including weekends. A qualified fitness specialist oversaw each session, providing guidance and motivation. The exercises were adapted from the multicomponent Vivifrail program [290], designed to prevent weakness and falls. The morning sessions included personalized progressive resistance exercises, balance training, and walking exercises. Resistance training was customized to each participant’s abilities using adjustable resistance machines, with a target of 2 to 3 sets of 8 to 10 repetitions at 30% to 60% of the participant’s 1-repetition maximum. These exercises focused on lower-body muscles (such as chair squats, leg presses, and knee extensions) and one upper-body exercise (seated chest press). Participants were instructed to perform the exercises at high speed to maximize muscle power, with attention given to correct technique. Balance and gait exercises increased in difficulty over time and included tasks like semi-tandem standing, line walking, step practice, navigating small obstacles, and proprioceptive exercises on unstable surfaces (using foam pads), along with altering the base of support and shifting weight from one leg to the other. In the evening, participants performed functional exercises without supervision, using light weights (0.5 to 1 kg anklets and a handgrip ball), such as knee extensions and flexions, hip abductions, and daily walks in the unit corridor, following the guidelines of the Vivifrail [290] program for clinical physical exercise.

The outcomes of these studies varied depending on the delivery and adherence to the interventions. A previous systematic review of exercise interventions for hospitalized older adults found that their impact on activities of daily living (ADL) performance was unclear. However, a small but significant reduction in hospital stay length and overall costs was reported [291]. For elective admissions, prehabilitation, involving increased physical activity prior to admission, may be beneficial. A randomized controlled trial comparing prehabilitation with rehabilitation in patients undergoing colorectal surgery showed similar complication rates between the groups but significantly improved 6 min walking test (6MWT) results in the prehabilitation group at the eight-week follow-up [292].

In addition, treating acute sarcopenia in the oral region through oral health management and tongue training provided by dental hygienists may improve oral intake, contributing to the overall management of acute sarcopenia [293].

Exercise can also have beneficial effects on gut microbiota composition [294]. Resistance training, in particular, can encourage the growth of beneficial bacteria such as *Faecalibacterium prausnitzii*, *Eubacterium*, *Roseburia*, and *Ruminococcus* species. These bacteria produce short-chain fatty acids (SCFAs), which act as an energy source for muscles. Administering SCFAs to germ-free mice, which typically experience muscle atrophy, helped reverse muscle deterioration and improved muscle strength [295].

Furthermore, professional athletes often exhibit greater gut microbiota diversity compared to sedentary individuals [296,297] and this diversity correlates with better physical fitness markers, such as peak oxygen uptake [298].

### 10.2. Nutritional Interventions

No randomized trial has specifically enrolled older hospitalized patients with sarcopenia to evaluate the effects of nutritional interventions so far [262]. However, nutritional counselling is crucial during acute conditions to prevent malnutrition [299].

#### 10.2.1. Proteins

Establishing protein needs and ensuring that protein intake is distributed across all meals and snacks is essential [300]. According to the PRO-TAGE recommendations [301], the required amount of protein during an acute condition depends on various factors: (i) the type and severity of the illness, (ii) the patient’s nutritional condition before becoming ill, and (iii) how the illness affects nutritional health. Typically, older adults dealing with acute disease need around 1.2 to 1.5 g of protein per kilogram of body weight (BW) per day. However, for those experiencing more serious conditions, injuries, or malnutrition, protein needs can increase up to 2.0 g/kg BW/day. An important exception applies to older individuals with severe kidney disease who are not undergoing dialysis, as they should reduce their protein intake (0.8 g/kg BW/day) to avoid further kidney strain.

#### 10.2.2. Leucine and Beta-Hydroxybeta-Methylbutyrate

Both leucine and beta-hydroxybeta-methylbutyrate (HMB) may help enhance muscle mass and muscle function [301], but more studies are needed to recommend precise dosages in the treatment of acute sarcopenia. Leucine intake is particularly important [302]. Leucine is a type of branched-chain amino acid. It stands out as one of the most effective in promoting anabolic activity compared to other essential amino acids [303,304]. A meta-analysis revealed that supplementing with leucine increased muscle mass in older individuals with sarcopenia compared to controls (mean difference of 1.14 kg, 95% CI (0.55, 1.74), *p* = 0.0002) [305].

HMB is an anabolic metabolite derived from leucine that is produced in muscles and occurs in small quantities in the diet. When taken orally, HMB promotes muscle protein synthesis in a manner comparable to leucine but reduces muscle protein breakdown more effectively than leucine [306].

As only 5% of leucine is metabolized to HMB, direct administration of HMB may therefore be a more efficient alternative [307]. HMB administration prevents loss of lean leg mass in healthy older adults during bed rest [308] and has been shown to reduce post-discharge mortality in hospitalized populations [309]. Moreover, a systematic review and meta-analysis, encompassing 15 randomized controlled trials (RCTs) across various clinical scenarios, revealed that HMB supplementation (either on its own or as part of combined formulations) contributed to increases in muscle mass (standard mean difference = 0.25; 95% CI: −0.00, 0.50; *z* = 1.93; *p* = 0.05) and strength (standard mean difference = 0.31; 95% CI: 0.12, 0.50; *z* = 3.25; *p* = 0.001; *I*^2^ = 0%), though with a small to moderate effect size [310].

In diverse patient groups, such as those undergoing orthopedic surgery [311] and geriatric [312], general [265,313], respiratory medicine [314,315], and critical care [316] patients, various interventions have been tested. These include protein-enriched foods [317,318], supplements [265,313,319], HMB [311], and nutritional consultation [289].

#### 10.2.3. Creatine Monohydrate

Creatine is an organic compound naturally produced in the kidneys and liver through chemical reactions involving the amino acids arginine, glycine, and methionine. It can also be obtained through external sources such as meat and fish and commercial supplements. About 95% of the body’s creatine is located in skeletal muscles, with roughly 66% stored as phosphocreatine (PCr). PCr is essential for regenerating and maintaining adenosine triphosphate (ATP) levels [320].

Creatine monohydrate is a dietary supplement which betters bioenergetics both at rest and during physical activity. Multiple studies have demonstrated that supplementing with creatine monohydrate (≥3 g/day), alongside a strength training regimen, can enhance muscle mass and improve functional performance in older adults [321,322].

However, its role in the treatment of acute sarcopenia remains to be explored.

#### 10.2.4. Polyunsaturated Fatty Acids

The primary bioactive omega-3 long-chain PUFAs are eicosapentaenoic acid (EPA) and docosahexaenoic acid (DHA). These fatty acids are found in seafood, fish oil, krill oil, and certain algal oils. EPA and DHA work by lowering the production of inflammatory eicosanoids, such as prostaglandins, thromboxanes, and leukotrienes, and serving as precursors for alternative compounds, including resolvins, protectins, and maresins, which help reduce inflammation [323]. This process ultimately leads to a decrease in the release of inflammatory cytokines. The anti-inflammatory and inflammation-resolving properties of EPA and DHA are important for both the prevention and treatment of muscle loss [323]. In vitro studies suggest that *n*-3 PUFAs help reduce muscle protein loss and cell death caused by cytokines with catabolic effects [324]. Regular supplementation with *n*-3 PUFA has been linked to increases in muscle mass and better physical performance in healthy older adults [325]. Additionally, cancer patients receiving omega-3 supplements have shown improvements in muscle composition [326]. A meta-analysis also highlighted the beneficial effects of *n*-3 PUFA supplementation on muscle mass (effect size 0.31, 95% CI 0.01 to 0.60) and lower-body strength (mean difference 0.47, 95% CI 0.02 to 0.93, *p* = 0.022) but not handgrip strength (effect size: 0.91, 95% CI: 1.13 to 2.96, *p* = 0.326) across both healthy individuals and those with clinical conditions [323].

#### 10.2.5. Nutritional Supplements

A subanalysis of the NOURISH trial [327] revealed that providing nutritional supplements (with 350 kcal, 20 g protein, 11 g fat, 44 g carbohydrate, 1.5 g calcium-HMB, and 26 other essential vitamins and minerals) led to an improvement in handgrip strength compared to placebo among acutely malnourished patients.

#### 10.2.6. Probiotics and Prebiotics

It is likely that acute conditions triggering acute sarcopenia may also affect gut microbiota composition. The development of an unfavorable microbiota profile, characterized by low diversity, an increase in opportunistic pathogenic bacteria, reduced expression of SCFA-related genes, and diminished sugar-decomposition capacity—along with enhanced proteolytic activity, as observed in older individuals with chronic inflammation [328]—has yet to be confirmed in cases of acute sarcopenia during acute inflammation.

Nevertheless, the administration of probiotics and prebiotics, which can improve intestinal dysbiosis, has been shown to have beneficial effects on muscle mass and function in chronic sarcopenia. This approach may also prove helpful in the treatment of acute sarcopenia.

Probiotics are live microorganisms that aid in restoring gut flora after stress and may benefit muscle health through several mechanisms. Firstly, probiotics can release proteases and peptidases, which enhance protein digestion and increase the availability of amino acids for muscle protein synthesis. Secondly, they help maintain gut integrity by reducing the leakage of bacterial cells and their proinflammatory toxins into the bloodstream, which are known to trigger inflammation and have catabolic effects on muscles [329]. Indeed, probiotic supplementation has been shown to improve markers of intestinal barrier integrity and reduce inflammation in both human studies [330] and rodent studies [331,332]. Thirdly, probiotic bacteria are known producers of short-chain fatty acids (SCFAs), which play a crucial role in muscle health. A strong association between SCFAs and muscle mass and strength has been demonstrated in both human studies [333] and murine models [295]

Supplementation with *Lactobacillus plantarum* TWK10 for six weeks has been shown to increase muscle mass and function in frail older adults [334], primarily by enhancing glycogen concentration in muscle tissue [335] and reducing inflammatory markers [336]. Analogously, in aged mice, *Lactobacillus casei* Shirota supplementation attenuates sarcopenia. The mechanisms behind this effect include the modification of gut microbiota composition to preserve SCFA levels, along with the reduction of mitochondrial dysfunction, reactive oxygen species, and inflammation [337].

Prebiotics have the potential to improve intestinal dysbiosis and, in some studies, have been shown to positively affect chronic sarcopenia [338]. As a result, they may also play a beneficial role in the treatment of acute sarcopenia by promoting a healthier gut environment and supporting muscle function.

Prebiotics are indigestible components of food that promote the growth of beneficial bacteria in the gut. Common prebiotic sources include chicory root, which provides inulin, and garlic and onions, which are rich in fructooligosaccharides (FOSs). Soybeans and lentils also serve as sources of galactooligosaccharides (GOSs). These compounds can enhance the production of short-chain fatty acids (SCFAs) by gut bacteria, which play an important role in maintaining gut health and muscle function.

In particular, studies have shown that supplementation with inulin and FOS can improve physical outcomes, such as grip strength in frail older adults [338] and endurance [339] in young healthy adults.

However, it is important to note that other studies have reported inconsistent results regarding the supplementation of prebiotics and probiotics [340,341] These variations suggest that their efficacy may depend on various factors such as dosage, population, and study design. Moreover, their potential effects on acute sarcopenia have not yet been thoroughly investigated, leaving a gap in our understanding of how these supplements may influence muscle health in the short term.

#### 10.2.7. Combined Approaches

Combined approaches involving nutritional consultation and strength/resistance training have also been explored [265,289,313]. These interventions aimed to enhance physical performance [265,313,316,317,318,319], strength [311,313,314,317,318,319], and muscle mass [265,315,342]. However, the small scale of these studies limits the generalizability of their results. Also, in patients with acute stroke, a high-energy diet combined with adequate rehabilitation time was linked to the prevention of acute sarcopenia [343]. Similarly, in older patients in convalescent hospitals, the combination of branched-chain amino acids and rehabilitation improved ADL and increased muscle mass [344].

### 10.3. Neuromuscular Electrical Stimulation

Neuromuscular electrical stimulation (NMES), which involves applying electrical currents to stimulate muscle contractions, has been explored as a potential intervention for acute sarcopenia, particularly when mobilization is not feasible, such as in ICU settings. However, trials using NMES have yielded conflicting results. A randomized controlled trial involving critically ill patients post-cardiothoracic surgery found no effect of bilaterally applied NMES on quadriceps muscle layer thickness, although patients in the NMES group regained muscle strength faster than the control group. The mean age in the NMES group was 63.3, compared to 69.7 in the control group [345]. Conversely, another study found that unilaterally applied NMES significantly prevented reductions in muscle fiber cross-sectional area compared to biopsies from control quadriceps, with a mean patient age of 70 [346]. In geriatric patients, a trial combining NMES with exercise did not show a statistically significant difference in gait speed changes between those receiving functional training alone and those receiving functional training with NMES [276]. In contrast, a trial in respiratory medicine patients showed significantly less decline in knee extension strength with NMES compared to usual care. Additionally, a trial in a general medicine population demonstrated significant improvements in physical performance in the group treated with NMES plus physical activity compared to the usual care group [347].

### 10.4. Pharmacological Treatments

At present, no medication has been approved for the treatment of acute sarcopenia [262].

Pharmaceutical interventions for treating acute sarcopenia have included growth hormone (GH) [348,349], testosterone [265], and erythropoietin (EPO) injections [350].

The study by Weissberger et al. [348] examined the effects of GH administration on muscle mass of patients undergoing hip replacement. GH was given both pre-operatively (for 14 weeks) and post-operatively (for one month, with doubled dose for first 2 weeks post-operatively).

Before surgery, lean body mass increased by an average of 5.2% (approximately 1.8 kg) in the group treated with GH compared to the placebo group (difference 4.9, 95% CI 0.3–9.6, *p* = 0.037) but decreased by 3% in both groups after surgery (difference −0.4, 95% CI −4.1–3.4, *p* = 0.84). GH treatment determined a significant improvement in the hip abductor strength on the non-operated side, where strength increased by 7%, compared to a 25% decrease in the placebo group (*p* < 0.02). Post-operatively, walking distance over four minutes improved by an average of 26.9 m in the GH group, whereas the placebo group saw a decline of 19.5 m (difference 46.4, 95% CI 1.3–91.5, *p* = 0.04). Most patients treated with GH experienced dose-dependent side effects. Signs of fluid retention, including hand swelling, carpal tunnel syndrome, and ankle edema, along with increased or newly appearing pain in joints other than the injured hip, were noted. These issues were mostly well tolerated and, in some instances, subsided without treatment.

Another pilot study by Hedström et al. [349] assessed the effect of GH given post-operatively in 20 hip fracture patients. The treatment lasted between 21 and 28 days, based on the length of the patient’s hospital stay. The patients in the group receiving GH treatment maintained their lean body mass throughout the treatment period, whereas the placebo group experienced a significant reduction in lean body mass (difference in lean body mass (g) from baseline to 4 weeks after surgery: −646 g in the GH group vs. −3246 g in the placebo group *p* = 0.03). However, the difference from baseline in lean mass two months after the termination of treatment was not significant anymore in the two groups (–3164 g in the GH group vs. −2856 g in the placebo group *p* = 0.5). Quadriceps strength did not differ in the GH and placebo group both immediately after the termination of treatment and two months later. In this study, two patients in the GH group developed soft edema in both feet that resolved after the reduction of the GH treatment dose to 50%.

In a pilot study conducted by Deer et al. [265] an intramuscular injection of testosterone enanthate within 24 h of hospital discharge (200 mg for men and 100 mg for women) improved physical performance (change in SBBP 4 weeks post-discharge: 2.83 ± 2.07 in the testosterone group vs. 1.31 ± 1.93 in the placebo group) and reduced 30-day readmission rates (5% in the testosterone group vs. 28% in the placebo group) in older patients discharged from an acute care unit. No adverse events related to testosterone treatment were reported in the study; however, only 19 individuals received testosterone, which may have led to an underestimation of potential side effects. Therefore, possible side effects—such as cardiovascular events, prostatic hyperplasia and accelerated growth of pre-existing prostate cancer, increased risk of blood clots, skin conditions like acne, fluid retention, mood and behavioral changes, and liver issues—should be carefully considered, and the risk–benefit ratio should be evaluated when considering testosterone as a treatment option for acute sarcopenia.

EPO has manifold effects. Beyond promoting blood cell production, it prevents cell death, reduces oxidative stress and inflammation [351,352], and promotes the growth and maturation of skeletal muscle myoblasts [353,354]. A study by Rotter et al. [355] showed that in mice intramuscular administration of EPO led to a significant enhancement in muscle strength by day 7 compared to saline-treated controls. Moreover, following injury, the proliferation of satellite cells and interstitial cells was significantly higher in EPO-treated mice compared to the control group. Another study by Hida et al. [356] showed that mice treated with EPO increased muscle strength and had a greater cellular regeneration compared to non-treated mice.

The beneficial effects of EPO were also confirmed in humans. In a study conducted by Zhang et al. [350], patients aged 60 and above undergoing hip surgery for femoral intertrochanteric fractures were given daily intravenous EPO injection (10,000 IU) for 10 days after surgery, while the control group received a corresponding amount of normal saline. Both groups received the same nutrition, diet, fluids, and exercise.

One week after surgery in females, handgrip strength increased more in the EPO-treated group than in the control group (13.95 ± 3.327 kg vs. 9.30 ± 2.812 kg, *p* < 0.05). This trend was confirmed both 2 and 4 weeks after surgery. In contrast, males showed no statistically significant difference in handgrip strength between the intervention and control groups.

In both female and males, treatment with EPO produced a small but significant reduction in muscle mass loss after orthopedic surgery (females before intervention: ASM/kg 12.77 ± 1.49 in those treated with EPO vs. 12.41 ± 1.23 in controls; 4 weeks after surgery: ASM/kg 12.98 ± 1.66 in treated vs. 12.48 ± 1.25 in controls; males before intervention: ASM/kg from 18.60 ± 1.67 in those treated with EPO vs. 18.37 ± 1.76 in controls; 4 weeks after surgery: ASM/kg 18.81 ± 1.82 in treated vs. 18.36 ± 1.85 in controls). No adverse effect of the treatment with EPO was reported.

## 11. Future Perspectives

While this review provides a comprehensive summary of the existing literature on acute sarcopenia, there are several major open questions that warrant further exploration. First, there remains a limited understanding of the precise molecular mechanisms driving rapid muscle loss following acute events such as trauma, surgery, or severe illness. Further research is needed to elucidate the exact pathways, including those related to inflammation, anabolic resistance, and hormonal dysregulation, to better inform targeted interventions. Second, although biomarkers such as C-reactive protein and GDF-15 have been associated with muscle wasting, their accuracy and clinical utility as early diagnostic tools in acute sarcopenia are still uncertain. Future studies should focus on validating these biomarkers and identifying additional indicators that can predict sarcopenia risk and recovery. Additionally, the therapeutic efficacy of interventions like nutritional supplementation, pharmacological agents, and physical therapies remains inconsistent across studies, indicating a need for larger, well-designed trials to establish standardized treatment protocols. Looking forward, personalized approaches that integrate genetic, metabolic, and clinical data offer a promising avenue for tailoring sarcopenia interventions to individual patient profiles. Moreover, the role of multidisciplinary care involving geriatricians, nutritionists, and physical therapists should be explored further, as collaborative care models could enhance patient outcomes. Lastly, advancements in technology, such as real-time muscle mass and function monitoring using muscle ultrasound or bioimpedance analysis, present an exciting opportunity for early detection and timely interventions. These areas represent critical frontiers in the management and understanding of acute sarcopenia and require attention in future research endeavors.

## 12. Conclusions

Acute sarcopenia is a significant and often underrecognized condition that can severely impact the recovery and overall health of hospitalized patients, particularly older adults. Despite advancements in understanding its pathophysiology, the development of effective interventions remains challenging. Current evidence highlights the potential benefits of early nutritional and physical activity interventions, although results have been mixed and further research is necessary to establish standardized treatment protocols.

Future studies should focus on the early identification of acute sarcopenia through the use of predictive biomarkers and consistent monitoring of muscle strength and size. These efforts, combined with a deeper understanding of the underlying mechanisms, will enable more precise risk stratification and targeted therapies. Additionally, raising awareness among healthcare providers about the importance of early intervention and the role of multidisciplinary teams in managing acute sarcopenia is crucial for improving patient outcomes. Overall, addressing acute sarcopenia proactively rather than reactively holds promise for enhancing recovery, reducing hospital stays, and improving the quality of life for affected individuals. The integration of comprehensive management strategies into routine clinical practice will be essential in mitigating the impacts of this condition.

## Figures and Tables

**Table 1:** Sarcopenia: cut-offs for the diagnosis.

	EWGSOP2		AWGS		SDOC	
Muscle strength	Grip strength	<27 kg ♂; <16 kg ♀	Grip strength	<28 kg ♂; <18 kg ♀	Grip strength	<35.5 kg ♂; <20 kg ♀
	5-time chair stand test	>15 s				
Muscle mass	ASM	<20 kg ♂; <15 kg ♀			Not required	
	ASM/height^2^	<7 kg/m^2^ ♂; <5.5 kg/m^2^ ♀	ASM/m^2^	<7 kg/m^2^ ♂; <5.4 kg/m^2^ ♀		
Muscle performance	Gait speed	≤0.8 m/s	Gait speed	≤1 m/s	Gait speed	<0.8 m/s
	SPPB	≤8 point score	SPPB	≤9 point score		
	TUG	≥20 s	5-time chair stand test	≥12 s		
	400 m walk test	Non-completion or ≥6 min for completion				
Sarcopenia definitions		Low strength + low muscle mass		Low strength OR low performance + low muscle mass		Low strength + low performance
	SEVERE:	Low strength + low muscle mass + low performance	SEVERE:	Low strength + low muscle mass + low performance		

AWGS = Asian Working Group for Sarcopenia, ASM = Appendicular Skeletal Muscle Mass, EWGSOP2 = European Working Group on Sarcopenia in Older People 2, SDOC = Sarcopenia Definitions and Outcomes Consortium, SPPB = Short Physical Performance Battery, TUG = Timed Up and Go Test, ♂ = male, ♀ = female.

**Table 2:** Potential biomarkers of acute sarcopenia.

Biomarker	Concentrations	Mechanisms
MicroRNA		
*miR-29b*	Elevated	Inhibits IGF-1 and PI3K, leading to muscle atrophy
*miR-542*	Elevated	Targets mitochondrial ribosomal proteins (S2, S10, S18C, S25, S26, and S27) inducing stress, leading to decreased expression of proteins encoded by mitochondrial DNA. This reduction is expected to impair mitochondrial function, including energy production. Moreover, it targets inhibitors of the TGF-b signaling pathway, which is known to mediate muscle atrophy, suggesting that it may increase TGF-b signaling
*miR-424*	Elevated	Reduces rRNA and protein synthesis in muscle cells
*miR-181a*	Reduced	Is an endogenous regulator of mitochondrial dynamics through concerted regulation of Park2, p62/SQSTM1, and DJ-1 in vitro. Downregulation of miR-181a with age was associated with an accumulation of autophagy-related proteins and abnormal mitochondria. Restoring miR-181a levels in old mice prevented accumulation of p62, DJ-1, and PARK2 and improved mitochondrial quality and muscle function
*miR-1*	Reduced	Helps maintain muscle homeostasis by downregulating Pax3, a transcription factor necessary for muscle progenitor cell activity. This downregulation is essential for initiating the myogenic program, which leads to the formation and differentiation of muscle cells. However, during acute sarcopenia, the dysregulation of miR-1 can impede the proper initiation of the myogenic program. This disruption can result in impaired muscle regeneration and repair, exacerbating muscle atrophy. Additionally, miR-1 is involved in other pathways critical for muscle function, including those related to muscle protein synthesis and degradation. Altered miR-1 expression can therefore lead to an imbalance in these pathways, further contributing to muscle wasting and weakness
*miR-133b*	Reduced	Regulates fundamental processes of myogenesis including myoblast differentiation, regeneration, and satellite cell fate determination. Its downregulation appears to promote satellite cell quiescence.
GDF-15	Elevated	Member of the transforming growth factor-beta (TGF-β) superfamily and is significantly upregulated in various forms of stress
FGF21	Promising role as acute sarcopenia biomarker to be confirmed in future studies	Produced in large quantities in response to muscular stress; reduces oxidative stress damage to skeletal muscle mitochondria; association with chronic sarcopenia

GDF-15: Growth Differentiation Factor 15; FGF21: Fibroblast Growth Factor 21.
